# Differential Prognostic Value of Residual Nodal Burden in Breast Cancer Subtypes

**DOI:** 10.21203/rs.3.rs-4810058/v1

**Published:** 2024-09-02

**Authors:** Christine Hong Ngoc Che Thai, Selena J. An, Conner R. Haase, Julia M Selfridge, Chris B. Agala, Kristalyn K. Gallagher, Philip M. Spanheimer

**Affiliations:** University of North Carolina at Chapel Hill; University of North Carolina at Chapel Hill; University of North Carolina at Chapel Hill; University of North Carolina at Chapel Hill; University of North Carolina at Chapel Hill; University of North Carolina at Chapel Hill; University of North Carolina at Chapel Hill

**Keywords:** ypN status, neoadjuvant chemotherapy, breast cancer subtypes

## Abstract

**Purpose:**

Residual cancer burden (RCB) index after neoadjuvant chemotherapy (NAC) is highly prognostic in patients with breast cancer (BC) but does not account for subtype or the precise impact of residual nodal burden (RNB). We aimed to precisely de ne the effect of RNB on survival by subtypes.

**Methods:**

Adult women with non-metastatic BC diagnosed from 2006–2021 in the National Cancer Database (NCDB) who received NAC followed by surgery within 8 months were included. RNB was also evaluated as a predictor of mortality with multivariable logistic regression. Kaplan-Meier analyses were performed to compare overall survival.

**Results:**

51,917 patients were included. After adjustment, ypN stage was the strongest predictor of mortality, with an odds ratio (OR) of 2.24 (95% CI 2.08–2.41) for ypN1 vs ypN0 and increased with increasing nodal burden - ypN2 vs ypN0 OR 5.03, 95% CI 4.60–5.51 and ypN3 vs ypN0 OR 8.85, 95% CI 7.88–9.93. Stratification of survival curves with higher RNB is most pronounced for triple-negative breast cancer (TNBC) with an absolute difference of 64% in 5-year overall survival between ypN0 and ypN3 patients, and lowest for the ER+/HER2− subtype with a 25% absolute difference in 5-year OS between ypN0 and ypN3 patients. On interaction analysis, ypN status was a stronger predictor of mortality for the TNBC subtype compared to other subtypes.

**Conclusion:**

RNB has a significantly different impact on survival by BC subtypes. Future study of optimal therapeutic strategies for patients with residual nodal disease after NAC should account for subtype specific differences in prognosis.

## Introduction

Breast cancer (BC) is categorized by receptor expression into four distinct subtypes: ER+/HER2−, ER+/HER2+, ER−/HER2+, and triple-negative breast cancer (TNBC), which generally correlate with molecular subtypes. BC subtypes have unique outcomes and response to therapeutics [, , ]. Pathologic complete response (pCR) to neoadjuvant chemotherapy (NAC), defined as ypT0N0, is associated with improved oncologic outcomes []. More recently, the residual cancer burden (RCB) index has been developed to further categorize patients with incomplete response into prognostic groups []. RCB is a score derived from several pathologic features – primary tumor bed area, overall cancer cellularity, percentage of cancer that is in situ disease, as well as number of positive lymph nodes, and diameter of largest lymph node metastasis. While RCB score is prognostic in all breast cancer subtypes, receptor subtype remains prognostic after accounting for RCB []. Currently, RCB (beyond pCR vs residual disease) is not used to guide treatment decisions.

Different receptor subtypes have different likelihood of nodal metastasis for a given T stage, with ER+/HER2− tumors being the most nodotropic. Further, rates of nodal clearance with NAC are highest for TNBC and HER2 + type tumors compared to ER+/HER2− type tumors. Based on distinct probability of nodal metastasis and distinct nodal response rates, we hypothesize that residual nodal burden (RNB) for each BC subtype is associated with distinct survival outcomes. Precise characterization of outcomes in patients with residual nodal disease by breast cancer subtype will identify opportunities to optimize therapeutic strategies based on mortality risk. We aim to compare survival outcomes of RNB after NAC between breast cancer subtypes and identify predictors of mortality in patients with RNB after NAC.

## Methods

### Cohort Selection

This study analyzed data collected in the National Cancer Database (NCDB). Adult women diagnosed with clinical stage 2–3 BC in 2006–2021 who received NAC followed by surgery within 8 months were included. Women with metastatic disease, had surgery first, died, or were lost in follow-up within 8 months of diagnosis were excluded. Data collected compared among four cohorts based on hormone receptor subtypes: ER+/HER2−, ER+/HER2+, ER−/HER2+, and TNBC. Nodal pCR, defined as post-treatment ypN0, and RNB, defined as post-treatment ypN+, were compared between these subtypes.

### Statistical Analysis

Patient demographics and clinical characteristics of patients and tumors were reported using descriptive statistics of median, interquartile ranges (IQRs) for continuous variables and number, percentages (n, %) for binary and categorical covariates. Adjusted logistical regression was used to identify factors associated with mortality among patients with clinically node-positive breast cancer and to assess interactions between subtypes ypN status. Results were presented in odds ratio (ORs) and 95% confidence intervals (CIs). Missing observations were excluded. Kaplan-Meier (KM) analysis was used to determine overall survival among different breast cancer subtypes, stratified by post-treatment ypN status. All analyses were conducted using Stata 18.5 (College Station, Texas). The study was approved by the Institutional Review Board at the University of North Carolina at Chapel Hill (IRB# 20–1493).

## Results

### Patient and Treatment Characteristics

In total, 51,917 patients were included in this study. There were 18,009 women diagnosed with ER+/HER2− BC, 3,325 with ER+/HER2+ BC, 7,958 with ER−/HER2+ BC, and 22,625 with TNBC (Table 1). The average age among the four cohorts was 53 years. The majority of patients were White, resided in a metropolitan/urban area, insured via private insurance, and had a Charlson Comorbidity Index (CCI) score of 0. Around half of the patients had AJCC clinical stage II vs stage III disease, and stage III disease was most prevalent in patients with TNBC (58.5%) while stage II disease most prevalent in the ER+/HER+ subtype (54.1%), p<0.001. The majority of patients presented with ductal histology and lobular histology was rare in ER− patients, and ER− patients were most commonly poorly differentiated.

The majority of patients (67.3%) underwent total mastectomy. Patients with TNBC were more likely to undergo partial mastectomy (36.1%) compared to patients with ER+/HER2− (28.2%) or ER+/HER2+ (29.7%) subtypes, p<0.001. In total, 23.9% of patients underwent sentinel lymph node biopsy (SLNB) without axillary dissection, and this was more commonly performed for the TNBC and ER−/HER2+ subtypes (30.4% and 31.5% respectively) compared to ER+/HER2+ (22.7%) and ER+/HER2− (20.1%). Adjuvant radiation therapy was delivered to the majority of patients, with the lowest delivery for ER+/HER2+ patients (65.7%).

### Predictors of Mortality in Patients Receiving NAC for cN+ Breast Cancer

Adjusted associations between clinical and demographic factors and overall survival (OS) are listed in Table 2. After adjustment, there remained a significant association between age and Black race and mortality (Table 2). Insured patients were less likely to die (OR 0.82, 95% CI 0.70–0.97, p=0.02) compared to uninsured patients, and patients treated at a community hospital was associated with mortality (OR 1.11, 95% CI 1.04–1.19) compared to an academic/research institution. After adjustment, higher CCI, as well as both clinical stage and tumor grade, were associated with mortality. Mortality was associated with TNBC receptor subtype with overlapping OR for the remaining 3 receptor subtypes. ypN status was the strongest predictor of mortality in ypN3 patients (OR 8.85, 95% CI 7.88 – 9.33) compared to ypN0 patients.

### Long-Term Oncologic Outcomes Stratified by ypN status

**Figure 1:**
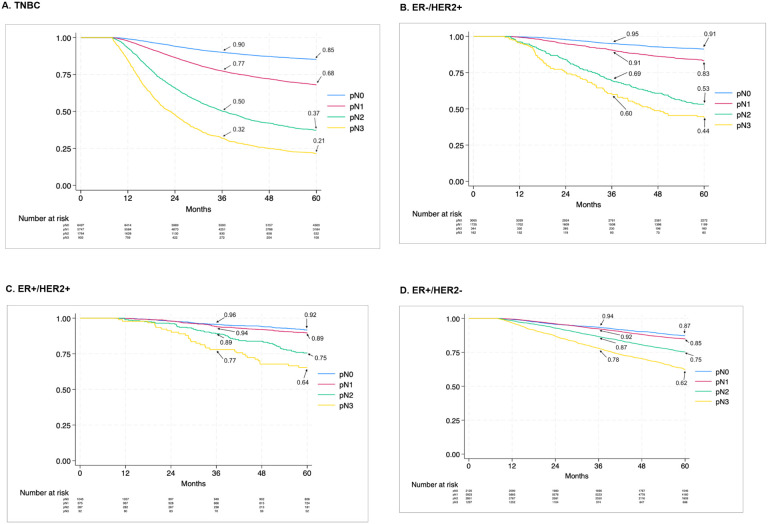
Kaplan-Meier analysis of overall survival among patients with clinically node-positive breast cancer receiving neoadjuvant chemotherapy, by subtype and ypN

Because nodal status was the strongest predictor of mortality, we performed KM analysis of overall survival stratified by ypN status for each receptor subtype. OS in patients with clinical positive lymph nodes after receiving NAC worsens with higher RNB for all subtypes. Specifically, for TNBC (Figure 1A), 5-year OS was 85% for patients with ypN0 disease and decreased with increasing residual nodal stage with 5-year OS of only 21% in patients with ypN3 disease, a 64% absolute difference. Similarly, a large absolute difference in 5-year OS was observed in the ER−/HER2+ subtype (Figure 1B), which was 91% in ypN0 patients and only 44% in ypN3 patients, a 47% absolute difference. In contrast, the absolute difference in 5-year OS was 28% between ypN0 and ypN3 patients with the ER+/HER2+ subtype (Figure 1C), and 25% (87% vs 62%) in patients with the ER+/HER2− subtype (Figure 1D).

### Interaction of ypN status and Receptor Subtype on Survival

**Figure 2:**
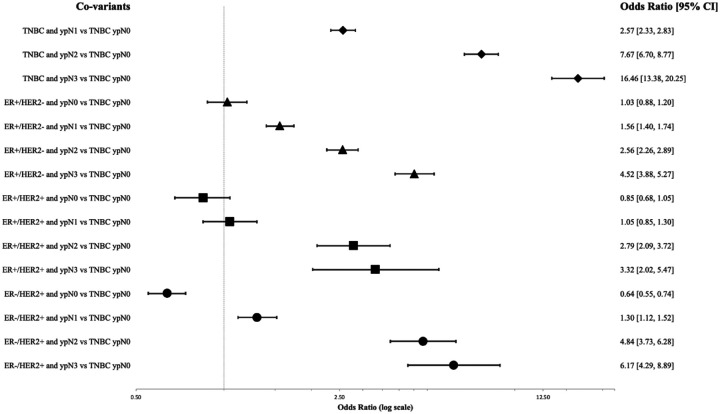
Forest Plot demonstrating the odds ratio for mortality of increasing nodal stage within each receptor subtype.

Stratification of KM curves by ypN stage was not uniform by receptor subtype, so we sought to more precisely delineate the interaction between post-treatment nodal stage and receptor subtype on overall survival. After adjustment for age, race, CCI, grade, and clinical stage, the impact of ypN stage on survival was assessed for each receptor subtype (Figure 2). The impact of ypN3 status (vs ypN0) was largest in the TNBC and ER−/HER2+ subtypes, OR 16.5 for TNBC and 9.6 for ER−/HER2+. The impact of increasing nodal stage on 5-year OS was least in the ER+/HER2− subtype.

## Discussion

Response to NAC is a strong predictor of outcomes in patients with BC. Classification of patients with residual disease by RCB score has further enhanced prognostication. While RCB score does include metrics of RNB, based on different likelihood of nodal metastasis and rates of nodal clearance with NAC between different receptor subtypes we sought to more precisely de ne the effect of RNB on survival by receptor subtype. Herein we show significant differences in 5-year OS in patients by residual nodal disease burden and receptor subtype. This illustrates significant differences in risk pro les by receptor subtype and nodal burden indicating that subtype-specific RNB could be a useful metric to consider when deciding how to prioritize adjuvant therapies.

Current guidelines recommend axillary lymph node dissection (ALND) and nodal field irradiation for all patients with any amount of residual nodal disease after NAC []. While ALND has not been shown to not improve recurrence free or OS in patients with low volume nodal disease in the upfront setting, concern exists that residual disease after neoadjuvant therapy is by definition treatment refractory and therefore may be more likely to lead to recurrence then treatment naïve nodal disease. Omission of ALND in patients with residual nodal disease is increasing, especially for patients with low volume residual disease, and we observed the lowest rates of completion ALND in TNBC and HER2 + subtypes which have the highest nodal clearance rates []. This is an area of active investigation and whether ALND can be safely omitted in patients with residual disease who receive nodal irradiation is currently being studied in the ALLIANCE 11202 randomized controlled trial [].

Response to chemotherapy measured by pCR vs residual disease to identify clinically meaningful differences in prognosis has been used to identify patients at higher risk for recurrence for escalated systemic therapy in the KATHERINE and CREATE-X trials [, ]. In those trials, patients with residual disease after NAC had improved recurrence free survival for HER2 + disease when treated with TDM-1 (KATHERINE) and for TNBC when treated with adjuvant capecitabine (CREATE-X). These trials demonstrate that treatment strategies using risk Stratification from response to preoperative chemotherapy can improve cancer outcomes.

Herein we identify a group of patients with ypN3 TNBC at significant risk for 5-year mortality. These findings demonstrate that the competing risk of death is high and that systemic therapies that have the potential to act on, and prevent or delay, metastatic disease should be prioritized. Recently, two antibody drug conjugates have shown promise for patients with TNBC – T-DXD for HER2-low disease and Sacituzimab Govitecan [, ]. As new systemic therapeutics are developed for patients with TNBC, patients with ypN3 disease represent an ideal patient population to study novel treatment strategies to improve recurrence and ultimately mortality.

In contrast to ypN3 TNBC patients, ypN3 ER+/HER2− patients have significantly better 5-year OS and less absolute difference from lower yp nodal stages. This could be due to biologically more indolent disease in the luminal intrinsic subtypes, but also the efficacy of systemic therapy and number of systemic therapy agents that can be used for long term disease control in the metastatic setting. Because the competing risk of mortality is lower for these patients, they may be more likely to derive benefit from loco-regional treatment, such as completion axillary dissection and nodal field irradiation. Future study of axillary de-escalation strategies should account for competing risks of metastatic disease and how that impacts the potential for isolated nodal recurrence.

Our study has several limitations. Our study was a retrospective analysis using NCDB data obtained through chart review across multiple different institutions. The variability in electronic medical records and users meant variability in data input, misclassification, and missing information. NCDB only captures information about a patient’s first course of treatment and does not include care following diagnosis and treatment of metastatic disease. Therefore, there is a lack of local, regional, and distant recurrence endpoints for each patient, which further limits our study’s ability to accurately correlate the true impact of RNB on OS. The lack of tumor cellularity, exact number of residually-positive lymph nodes, and size of the largest residual nodal metastasis in NCDB also limited our ability to calculate and compare our Stratifications to RCB index. In addition, participation in NCDB is completely voluntary. NCDB collects data from American College of Surgeons Commission on Cancer (CoC) accredited cancer programs, which can attribute to selection bias. Although CoC accreditation is a high quality metric, many large, well-perceived institutions are not accredited and therefore did not contribute to NCDB []. Results from our study may not fully be generalized to the general BC population in the United States. Although NCDB captures over 70% of cancer cases, 75.3% of cohort was White and 24.3% was Black. This may not be representative of the diverse population in the United States. Current and prospective clinical trials are necessary to determine the true causal effects of RNB on survival by BC subtypes as well as to determine the true effects of health disparities on RNB and OS in breast cancer patients.

## Conclusion

Measures of residual disease after NAC for patients with BC are associated with prognosis, but using these metrics for treatment decisions has not progressed beyond pCR vs RD. Our study focuses on more precisely defining the relationship between RNB and receptor subtype and effects on survival. Herein we demonstrate that RNB has a significantly different impact on survival by BC subtypes. TNBC patients with high RNB have poor OS, indicating that systemic treatment should be prioritized. Future study of optimal therapeutic strategies and treatment prioritization should account for RNB and subtype specific differences in prognosis.

## Figures and Tables

**Table 1.** Demographic and clinical characteristics of patients with clinically node positive breast cancer receiving neoadjuvant chemotherapy, by subtype

Characteristics	TNBC (n=22,625)	ER+/HER2+ (n=3,325)	ER+/HER2− (n=18,009)	ER−/HER2+ (n=7,958)	P value
Age (median in years, interquartile range)^a^	53 (21–90)	53 (23–90)	53 (21–90)	54 (23–90)	**<0.001**
Race, no (%)					
White	15,445 (72.3%)	2,532 (83.1%)	13,535 (81.0%)	5,883 (81.0%)	**<0.001**
Black	5,823 (27.3%)	500 (16.4%)	3,091 (18.5%)	1,356 (18.7%)	
Other	90 (0.4%)	15 (0.5%)	83 (0.5%)	25 (0.3%)	
Place of residence, no (%)					
Metro/Urban	22,316 (98.6%)	3,280 (98.7%)	17,760 (98.6%)	7,855 (98.7%)	0.98
Rural	309 (1.4%)	45 (1.4%)	249 (1.4%)	103 (1.3%)	
Insurance status, no (%)					
Not insured	751 (3.3%)	103 (3.1%)	613 (3.4%)	270 (3.4%)	0.82
Insured	21,874 (96.7%)	3,222 (96.9%)	17,396 (96.6%)	7,688 (96.6%)	
Treatment facility region, no (%)					
South	8,262 (43.0%)	1,214 (42.6%)	6,704 (43.2%)	2,877 (41.5%)	0.53
Northeast	3,295 (17.1%)	546 (19.2%)	2,700 (17.4%)	1,327 (19.1%)	
Midwest	4,864 (25.3%)	586 (20.6%)	3,581 (23.1%)	1,640 (23.7%)	
West	2,809 (14.6%)	504 (17.7%)	2,542 (16.4%)	1,088 (15.7%)	
Facility type, no (%)					
Academic/Research	6,488 (33.7%)	910 (31.9%)	4,961 (32.0%)	2,209 (31.9%)	0.05
Community	8,383 (43.6%)	1,381 (48.5%)	7,080 (45.6%)	3,160 (45.6%)	
Integrated Network	4,359 (22.7%)	559 (19.6%)	3,486 (22.5%)	1,563 (22.6%)	
CCI^a^, no (%)					
0	19,398 (85.7%)	2,925 (88.0%)	15,648 (86.9%)	6,919 (86.9%)	**<0.001**
1	2,534 (11.2%)	315 (9.5%)	1,846 (10.3%)	811 (10.2%)	
>1	693 (3.1%)	85 (2.6%)	515 (2.9%)	228 (2.9%)	
Clinical Stage, no (%)					
Stage 2	9,393 (41.5%)	1,800 (54.1%)	9,531 (52.9%)	4,028 (50.6%)	**<0.001**
Stage 3	13,232 (58.5%)	1,525 (45.9%)	8,478 (47.1%)	3,930 (49.4%)	
Histology, no (%)					
Ductal	20,380 (90.1%)	2,896 (87.1%)	14,224 (79.0%)	7,174 (90.2%)	**<0.001**
Lobular	236 (1.0%)	104 (3.1%)	1,739 (9.7%)	87 (1.1%)	
Infiltrating ductal	532 (2.4%)	176 (5.3%)	1,210 (6.7%)	223 (2.8%)	
Other	1,477 (6.5%)	149 (4.5%)	836 (4.6%)	474 (6.0%)	
Grade, no (%)					
Well differentiated	132 (0.6%)	102 (3.4%)	1,268 (7.7%)	68 (0.9%)	**<0.001**
Moderately differentiated	2,706 (12.9%)	1,131 (37.9%)	7,701 (46.6%)	1,676 (23.3%)	
Poorly differentiated	18,085 (86.4%)	1,752 (58.7%)	7,559 (45.7%)	5,453 (75.8%)	
Type of surgery, no (%)					
Partial mastectomy	8,169 (36.1%)	985 (29.7%)	5,076 (28.2%)	2,568 (32.3%)	
Total mastectomy	14,402 (63.7%)	2,322 (70.0%)	12,890 (71.7%)	5,371 (67.6%)	
None	32 (0.1%)	11 (0.3%)	24 (0.1%)	11 (0.1%)	**<0.001**
Lymph node surgery, no (%)					
SLNB^b^ only	6,247 (30.4%)	627 (22.7%)	3,273 (20.1%)	2,277 (31.5%)	**<0.001**
ALND^c^ only	10,464 (51.0%)	1,622 (58.8%)	8,911 (54.8%)	(3,723 (51.5%)	
SLNB and ALND	3,816 (18.6%)	508 (18.4%)	4,084 (25.1%)	1,224 (16.9%)	
Radiation therapy, no (%)					
No	4,469 (19.8%)	977 (29.4%)	3,106 (17.3%)	1,987 (25.0%)	**<0.001**
Yes	17,417 (77.0%)	2,184 (65.7%)	14,266 (79.2%)	5,683 (71.4%)	
Unknown	739 (3.3%)	164 (4.9%)	637 (3.5%)	288 (3.6%)	

a Charlson Comorbidity Index

b Sentinel lymph node biopsy

c Axillary lymph node dissection

**Table 2.** Adjusted logistic regression of factors associated with mortality among patients with clinically node-positive breast cancer after neoadjuvant chemotherapy

	OR^a^	95% CI^b^	P Value
Age, in years	1.02	[1.02 – 1.03]	**<0.001**
Race (ref: white)
Black	1.17	[1.09 – 1.25]	**<0.001**
Other	1.40	[0.88 – 2.24]	0.16
Place of residence (Metro/Urban as reference)
Rural	1.17	[0.92 – 1.48]	0.20
Insurance status (Not insured as reference)
Insured	0.82	[0.70 – 0.97]	**0.020**
Treatment facility region (South as reference)
Northeast	0.94	[0.86 – 1.02]	0.12
Midwest	1.00	[0.93 – 1.07]	0.97
West	0.92	[0.84 – 1.01]	0.08
Facility type (Academic/Research as reference)
Community	1.11	[1.04 – 1.19]	**0.003**
Integrated Network	1.08	[1.00 – 1.17]	0.06
CCI^c^ (0 as reference)
1	1.18	[1.08 – 1.29]	**<0.001**
>1	1.88	[1.60 – 2.20]	**<0.001**
Histology (Ductal as reference)
Lobular	1.20	[1.04 – 1.38]	**0.011**
Other	1.16	[1.05 – 1.27]	**0.002**
Grade (1 as reference)
2	1.49	[1.25 – 1.78]	**<0.001**
3	2.07	[1.73 – 2.46]	**<0.001**
Clinical stage (ref: stage 2)			
Stage 3	1.72	[1.63 – 1.83]	**<0.001**
Subtype (ref: TNBC^d^)			
ER+/HER2+	0.50	[0.44 – 0.57]	**<0.001**
ER+/HER2−	0.55	[0.51 – 0.59]	**<0.001**
ER−/HER2+	0.55	[0.50 – 0.61]	**<0.001**
ypN (ref: ypN0)			
ypN1	2.24	[2.08 – 2.41]	**<0.001**
ypN2	5.03	[4.60 – 5.51]	**<0.001**
ypN3	8.85	[7.88 – 9.93]	**<0.001**

a Odds ratio

b Confidence interval

c Charlson comorbidity index

d Triple negative breast cancer
